# Acute Coronary Syndrome revisited

**DOI:** 10.1186/1757-7241-21-S2-A16

**Published:** 2013-09-09

**Authors:** Niels Christian Kromann, Anne Grethe Mølbak, Jacob Hansen-Schwartz

**Affiliations:** 1 Emergency Dept., Køge Hospital, Denmark

## Background

Chest pain is an indicator of possible myocardial infarction. Definite diagnosis of non ST-elevation myocardial infarction (NSTEMI) requires sequential measurement of troponin levels. It is a challenge for the Emergency Department physician to select patients for observation. The aim of the present study was retrospectively to describe the patient cohort selected for observation with an extended focus on patients having the diagnosis confirmed.

## Methods

Patients admitted for observation for myocardial infarction at Køge Hospital in the period July to December 2012 were identified through “Landspatientregistret”. 273 consecutively admitted patients were identified.

Parameters recorded: Age, gender, date of admittance, and troponin levels. For patients with elevated troponin levels the following parameters were identified: Risk factors such as hypertension, diabetes mellitus, hypercholesterolemia, smoking, genetic disposition, and history of ischemic heart disease. Presence of novel ischemic ECG changes was registered as well as flow limiting lesions observed on performing coronary arteriography (CAG).

## Results

36 patients (13%) had NSTEMI confirmed. Mean age at time of admittance was 70 years (range 44 to 95 years). M:F gender distribution was 56:44. In comparison, mean age of patients not harbouring myocardial infarction was 63 years (range 27 to 96 years) with a gender distribution of 43:57. The age difference was statistically significant (p<0.05).

19 patients (53%) had two or more risk factors. Nine patients (25%) had ischemic ECG changes. 27 patients (75%) had CAG performed, of these 21 were pathological.

## Conclusion

Absence of ischemic ECG changes at the time of admittance is not a good predictor of a non-ischemic event. Presence of ischemic risk factors at the time of admittance increases the likelihood of an ischemic event. Absence of risk factors is an invalid predictor of non-ischemic events. The proportion of patients with non-ischemic chest pain is a differential diagnostic problem. Through further stratification it is the intention to take into account patients with unstable angina.

